# Vascular Access Thrombosis Events in Patients With Dialysis-Dependent CKD Treated With Vadadustat or Darbepoetin Alfa: The INNO_2_VATE Trial Program

**DOI:** 10.1016/j.xkme.2025.100997

**Published:** 2025-03-19

**Authors:** Wolfgang C. Winkelmayer, Steven K. Burke, Glenn M. Chertow, Kai-Uwe Eckardt, Wenli Luo, Todd Minga, Mark J. Sarnak, Prabir Roy-Chaudhury

**Affiliations:** 1 Section of Nephrology, Baylor College of Medicine, Houston, TX; 2 Akebia Therapeutics Inc, Cambridge, MA; 3 Stanford University School of Medicine, Stanford, CA; 4 Department of Nephrology and Medical Intensive Care, Charité - Universitätsmedizin Berlin, Berlin, Germany; 5 Division of Nephrology, Tufts Medical Center, Tufts University School of Medicine, Boston, MA; 6 Division of Nephrology and Hypertension, University of North Carolina Kidney Center, NC, and WG (Bill) Hefner VA Medical Center, Salisbury, NC

**Keywords:** Anemia, chronic kidney disease, hemodialysis, vadadustat, vascular access thrombosis

## Abstract

**Rationale & Objective:**

In the global phase 3 INNO_2_VATE program of patients with dialysis-dependent chronic kidney disease (DD-CKD) and CKD-related anemia (2 trials: patients new to [NCT02865850] and established on maintenance dialysis [NCT02892149]), vadadustat was noninferior to the erythropoiesis-stimulating agent darbepoetin alfa for cardiovascular safety and hemoglobin efficacy. Here, we investigated between-group differences in positively adjudicated vascular access thrombosis (VAT) events.

**Study Design:**

Phase 3, global, open-label, randomized, active-controlled clinical trials.

**Setting & Participants:**

A total of 3,902 patients who initiated dialysis within the past 16 weeks (incident DD-CKD trial; N = 365) or who had been treated with dialysis for >12 weeks (prevalent DD-CKD trial; N = 3,537).

**Intervention:**

Eligible patients randomized 1:1 to vadadustat or darbepoetin alfa.

**Outcomes:**

Positively adjudicated VAT events.

**Results:**

In the INNO_2_VATE program, at baseline, 3,590 (92.0%) randomized patients were receiving hemodialysis: 2,709 (69.4%) via an arteriovenous fistula and 340 (8.7%) via an arteriovenous graft. During 6,468 patient-years (PY) of follow-up, there were 426 positively adjudicated VAT events in 266 individual patients, with 146 randomized to vadadustat and 120 randomized to darbepoetin alfa. Corresponding first VAT rates were 4.79 and 3.86 per 100 PY, respectively (rate difference, 0.94; 95% CI, −0.10 to 1.97; hazard ratio [HR], 1.27; 95% CI, 0.99-1.62). When considering first and recurrent events, VAT rates were 6.58 and 6.59 per 100 PY for the vadadustat and darbepoetin alfa groups, respectively (rate difference, −0.01; 95% CI, −1.26 to 1.24; HR, 1.00; 95% CI, 0.83-1.21).

**Limitations:**

Trials were not specifically designed to assess VAT rates; uncertain generalizability to nontrial populations.

**Conclusions:**

In this secondary analysis of the INNO_2_VATE program in patients with DD-CKD and CKD-related anemia receiving hemodialysis, first VAT rates were numerically higher among patients treated with vadadustat versus darbepoetin alfa but statistically not different. The rates of first and recurrent VAT events were similar between treatment groups.

## Plain-Language Summary

The phase 3 INNO_2_VATE program studied patients with dialysis-dependent chronic kidney disease (CKD) and CKD-related anemia in 2 trials: one enrolled patients new to dialysis (365 patients) and another studied patients who had been treated with dialysis for at least 3 months (3,537 patients). In terms of cardiovascular safety and ability to treat CKD-related anemia, vadadustat and darbepoetin alfa were not meaningfully different. This analysis also looked at how many patients had dialysis access blockage. There were 146 patients with dialysis access blockages in the vadadustat group and 120 in the darbepoetin alfa group. Although the vadadustat group had a marginally higher rate of first blockages, when subsequent dialysis access blockages were also considered, the rates were similar between treatments.

## Introduction

Anemia is a hallmark complication of advanced kidney disease and is present in nearly all patients with kidney failure receiving maintenance dialysis.^1^^,^^2^ The pathophysiology of chronic kidney disease (CKD)-related anemia is complex, and contributing causes include defective oxygen sensing and dysfunctional feedback loops that can lead to inadequately low erythropoietin production, release, and hematopoietic response.^3^^,^^4^ The pillars of contemporary treatment of CKD-related anemia include erythropoiesis-stimulating agents (ESAs), intravenous iron administration, and, when required, red blood cell transfusions.^5^, ^6^, ^7^ However, treatment with ESAs, particularly when used to achieve higher versus lower hemoglobin concentrations, has been associated with cardiovascular safety concerns.^8^, ^9^, ^10^ In the United States, package inserts for all ESAs contain boxed warnings highlighting the potential for increased cardiovascular risk.^11^^,^^12^ Although most attention has been focused on the excess risks of heart failure and stroke,^8^, ^9^, ^10^ ESAs have also been associated with increased risk of venous thromboembolic disease. Further, more intensive use of ESAs has been associated with excess rates of hemodialysis vascular access thrombosis (VAT).^13^ The US Normal Hematocrit Cardiac Trial compared treatment with epoetin alfa with hematocrit targets of 42% versus 30% in patients with kidney failure and heart failure or ischemic heart disease receiving maintenance hemodialysis.^8^ This trial showed a significantly higher proportion of patients in the higher hematocrit target group experienced a VAT event (39% vs 29% in the lower target group; *P* = 0.001).^8^

Recent developmental programs for hypoxia-inducible factor prolyl hydroxylase inhibitors (HIF-PHIs), which exploit a novel pathway to treat CKD-related anemia, have also focused on VAT as a key safety event.^14^ HIF is a transcription factor that regulates the expression of various hypoxia-sensitive genes, including *EPO*, which serves as a key stimulus of erythropoiesis.^4^ HIF is regulated by oxygen-dependent proteasomal degradation, with a family of prolyl hydroxylases serving as oxygen sensors.^4^ HIF-PHIs constitute a recently developed class of compounds that stabilize HIF, promoting *EPO* expression, and, as a result, have the potential to ameliorate CKD-related anemia.^15^

Vadadustat is an oral HIF-PHI currently approved in 37 countries for use in patients with dialysis-dependent (DD) CKD, including in Japan,^16^ the European Union,^17^ and Australia.^18^ In the United States, vadadustat has been approved for patients with DD-CKD who have been treated with dialysis for at least 3 months.^19^ The exclusion of patients with <3 months of dialysis treatment was because of the US Food and Drug Administration’s conclusion that the risk of a major adverse cardiovascular event (MACE) had not been sufficiently studied in patients recently initiating dialysis.^20^^,^^21^ In a phase 3 program focused on patients receiving maintenance dialysis (INNO_2_VATE), vadadustat was shown to be noninferior to the ESA darbepoetin alfa in terms of cardiovascular safety (the primary endpoint, a composite of nonfatal myocardial infarction, nonfatal stroke, and all-cause mortality) and hematologic efficacy (achieved hemoglobin concentrations).

We conducted the present analysis to compare relative rates of VAT in patients with DD-CKD and CKD-related anemia randomized to vadadustat versus darbepoetin alfa.

## Methods

### Study Design

The present analysis is specific to patients with CKD-related anemia in the INNO_2_VATE clinical trials program, which consisted of 2 phase 3, global, randomized, open-label, sponsor-blind, active-controlled, noninferiority trials to compare the safety and efficacy of vadadustat with that of darbepoetin alfa in patients with kidney failure treated with dialysis. Specifically, the INNO_2_VATE clinical trials program was composed of a trial in patients who had only recently (within 16 weeks) initiated dialysis (incident DD-CKD trial, ClinicalTrials.gov identifier NCT02865850) and a trial in patients who had been receiving dialysis for >12 weeks (prevalent DD-CKD trial, ClinicalTrials.gov identifier NCT02892149). Details regarding the rationale, trial design, and primary safety and efficacy results of the INNO_2_VATE phase 3 trials have been published previously.^21^^,^^22^

Both trials were performed in compliance with the International Council for Harmonisation, in accordance with Good Clinical Practice guidelines and applicable local regulatory requirements and laws, and in line with the principles of the Declaration of Helsinki. Institutional review board approval was obtained at each participating center. All patients provided written informed consent before enrollment.

### Study Population

In both INNO_2_VATE trials, eligible patients were aged ≥18 years with DD-CKD and anemia with hemoglobin concentrations of 8-11 g/dL (incident DD-CKD trial) and 8-11 g/dL (United States) or 9-12 g/dL (non−United States) (prevalent DD-CKD trial). Patients had serum ferritin concentrations ≥100 ng/mL and transferrin saturation of ≥20% and had not received a red blood cell transfusion within 8 weeks before randomization. Patients were excluded if anemia was thought to be because of causes other than CKD or if they had uncontrolled hypertension or a recent cardiovascular event.

### Study Procedures

Eligible participants were randomized 1:1 to receive open-label vadadustat or darbepoetin alfa, stratified by geographic region (United States/Europe/Other Countries), New York Heart Association Functional Classification (0/I vs II/III), and hemoglobin concentration at entry (incident DD-CKD trial, <9.5 vs ≥9.5 g/dL; prevalent DD-CKD trial, <10 vs ≥10 g/dL).

The trials had the following 4 defined periods: (1) a correction or conversion period (weeks 0-23); (2) a maintenance period (weeks 24-52), which comprised the primary (weeks 24-36) and secondary (weeks 40-52) efficacy periods; (3) a long-term safety period (weeks 53 to end of treatment [182 weeks]); and (4) a 4-week safety follow-up period after the end of treatment.

The vadadustat starting dose for all participants was 300 mg orally once daily (2 150-mg tablets), with doses of 150, 450, and 600 mg available for adjustment to a maximum daily dose of 600 mg. Darbepoetin alfa was administered subcutaneously or intravenously, with the initial dose based on dose before randomization for those patients already receiving darbepoetin alfa or based on the local product label for participants not receiving darbepoetin alfa before randomization. We titrated doses of vadadustat and darbepoetin alfa to maintain target hemoglobin concentrations (10-11 g/dL in the United States; 10-12 g/dL in all other countries). We encouraged iron supplementation to maintain serum ferritin concentrations of ≥100 ng/mL or transferrin saturation ≥20%. We measured hemoglobin concentrations every 2 weeks for weeks 0-12 and every 4 weeks for weeks 12-52. From week 53 through the study end, we continued to monitor hemoglobin to determine if the dose of study medication should be adjusted, interrupted, or maintained to encourage maintenance of hemoglobin concentrations within the geography-specific target range. Dose adjustments were based on dose adjustment algorithms, investigator’s clinical discretion, incorporating the protocol guidance and considering the participant’s clinical condition, hemoglobin rate of increase, hemoglobin rate of decline, hemoglobin variability, and ESA responsiveness.

### Endpoints

The primary safety endpoint of the INNO_2_VATE trials was time to first MACE, prespecified as a pooled event−driven analysis.^22^ An Endpoint Adjudication Committee (EAC) adjudicated all potential cardiovascular events, blinded to participant treatment assignment, and adjudication outcomes were not provided to investigators during the course of the studies. The formal cardiovascular safety analyses only included events positively adjudicated by the EAC (ie, they confirmed that the specified endpoint met definitions). Among adjudicated cardiovascular events, thromboembolic events included arterial thrombosis, deep vein thrombosis, pulmonary embolism, and VAT.

The present analyses focus on positively adjudicated VAT events. Dialysis modality (hemodialysis vs peritoneal dialysis) and type of vascular access used for hemodialysis (arteriovenous fistula [AVF], arteriovenous graft [AVG], and catheter), in addition to the dialysis frequency, were ascertained at baseline and every 2 weeks for weeks 0-12 and every 4 weeks for weeks 12-52. Information pertaining to vascular access was not collected systematically during the 4-week safety follow-up period after the end of treatment; hence, the present analyses include data from the overall safety population, with sensitivity analyses in subpopulations as described below.

### Follow-up Time

We used an intent-to-treat framework. We followed patients from the time when the first patient signed the informed consent form through to the last visit of the last patient. Patients who did not experience MACE by study closure were censored on the date of their last study assessment. The main analysis focused on time to first VAT event, with a companion analysis including first and recurrent events. For the outcome of time to first VAT event, we did not censor for an (earlier) occurrence of arterial thrombosis, deep vein thrombosis, or pulmonary embolism.

### Statistical Analyses

We performed all analyses using the pooled safety populations, which included all randomized participants who received ≥1 dose of study drug irrespective of dialysis modality and vascular access at baseline. We summarized continuous variables using mean ± standard deviation or median (25th-75th percentile), where appropriate, and categorical variables using proportions.

First and recurrent VAT event data are reported as event counts (no.), follow-up time (patient-years [PY]), and event rates (no./100 PY); comparative effect measures are expressed as rate differences (no./100 PY) and hazard ratios (HRs) with the corresponding 95% CIs, where appropriate. Rate differences were calculated using the Cochran-Mantel-Haenszel method, and HRs were calculated using a stratified Cox regression model to compare the cumulative incidence of VAT events. All analyses were stratified by study and included covariates of baseline hemoglobin concentration, geographic region (United States vs Europe vs Other Countries), New York Heart Association Functional Classification (0 or I vs II or III), sex, age (≤65 years vs >65 years), race (White or non-White), history of cardiovascular disease (yes or no), and diabetes mellitus (yes or no).

### Sensitivity Analyses

While the main analyses used all patients in the safety population irrespective of baseline dialysis modality or vascular access, we conducted sensitivity analyses in which the cohort was restricted to (1) patients who had an AVF or AVG at baseline; (2) patients with an AVF at baseline; and (3) patients with an AVG at baseline.

## Results

The safety population of the INNO_2_VATE program included 3,902 patients, with 1,947 in the vadadustat group and 1,955 in the darbepoetin alfa group. Baseline characteristics for each group are shown in Table 1. When combined, 3,590 (92.0%) randomized patients were receiving hemodialysis at baseline; 2,709 (69.4%) had an AVF, and 340 (8.7%) had an AVG. In addition to an AVF or AVG, 850 (21.8%) patients also had another form of vascular access, such as a temporary or tunneled dialysis catheter. The median (25th-75th percentile) dialysis “vintage” was 2.3 (0.9-5.0) years, with 472 (12.1%) having initiated dialysis within 16 weeks before screening. The median (25th-75th percentile) duration of follow-up was 1.2 (0.8-1.7) years in the incident DD-CKD trial and 1.7 (1.2-2.2) years in the prevalent DD-CKD trial.

**Table 1:** Selected Demographic, Clinical, and Laboratory Characteristics of Patients at Baseline (Safety Population and Safety Population With Baseline Dialysis Type as AVF or AVG)

Characteristic	Safety Population		AVF or AVG at Baseline	
	Vadadustat N = 1,947	Darbepoetin Alfa N = 1,955	Vadadustat N = 1,525	Darbepoetin Alfa N = 1,524
**Demographics and baseline characteristics**				
Age, y, mean (SD)	57.8 (14.0)	58.1 (13.9)	58.0 (13.9)	58.6 (13.7)
Male, n (%)	1,089 (55.9)	1,110 (56.8)	866 (56.8)	872 (57.2)
Race, n (%)				
Asian	88 (4.5%)	106 (5.4%)	46 (3.0%)	60 (3.9%)
Black or African American	470 (24.1%)	478 (24.5%)	376 (24.7%)	384 (25.2%)
White	1,255 (64.5%)	1,231 (63.0%)	1,015 (66.6%)	973 (63.8%)
Not reported	51 (2.6%)	53 (2.7%)	38 (2.5%)	40 (2.6%)
Other	83 (4.3%)	87 (4.5%)	50 (3.3%)	67 (4.4%)
Region, n (%)				
United States	1,180 (60.6%)	1,181 (60.4%)	942 (61.8%)	931 (61.1%)
Europe	277 (14.2%)	295 (15.1%)	219 (14.4%)	242 (15.9%)
Other Countries	490 (25.2%)	479 (24.5%)	364 (23.9%)	351 (23.0%)
Hemoglobin (g/dL)				
Mean (SD)	10.2 (0.9)	10.1 (0.9)	10.2 (0.9)	10.2 (0.9)
Median	10.2	10.2	10.3	10.3
Q1, Q3	9.7, 10.8	9.6, 10.8	9.7, 10.8	9.7, 10.8
BMI (kg/m^2^), mean (SD)	28.5 (7.1)	28.4 (7.1)	28.5 (7.1)	28.4 (7.0)
NYHA Functional Classification, n (%)				
0 (no CHF) or I	1,698 (87.2%)	1,700 (87.0%)	1,333 (87.4%)	1,321 (86.7%)
II or III	249 (12.8%)	255 (13.0%)	192 (12.6%)	203 (13.3%)
Disease history, n (%)				
Diabetes mellitus	1,070 (55.0%)	1,088 (55.7%)	822 (53.9%)	835 (54.8%)
Cardiovascular disease^a^	934 (48.0%)	1,001 (51.2%)	760 (49.8%)	815 (53.5%)
VAT	191 (10.7%)	187 (10.5%)	N/A	N/A
**Dialysis-related characteristics**				
Time since dialysis initiation^b^				
Mean (SD)	3.7 (4.0)	3.6 (4.0)	4.1 (4.2)	4.0 (4.1)
Median	2.3	2.2	2.8	2.7
Q1, Q3	0.9, 5.0	0.8, 4.9	1.2, 5.8	1.2, 5.6
Incident dialysis patient^c^, n (%)	222 (11.4)	250 (12.8)	106 (7.0)	123 (8.1)
Dialysis modality, n (%)				
Hemodialysis	1,793 (92.2%)	1,797 (92.0%)	1,515 (99.3%)	1,520 (99.7%)
Peritoneal dialysis	152 (7.8%)	157 (8.0%)	10 (0.7%)	4 (0.3%)
Vascular access, n (%)				
AVF	1,365 (70.1%)	1,344 (68.7%)	1,365 (89.5%)	1,344 (88.2%)
AVG	160 (8.2%)	180 (9.2%)	160 (10.5%)	180 (11.8%)
Temporary catheter	43 (2.2%)	46 (2.4%)	0	0
Tunneled dialysis catheter	234 (12.0%)	226 (11.6%)	2 (0.1%)^d^	2 (0.1%)^d^
Other	143 (7.3%)	158 (8.1%)	0	0
Missing	2 (0.1%)	1 (0.1%)	0	0
Baseline ESA use^e^				
N	1,758	1,766	1,436	1,427
Epoetin, n (%)	965 (54.9%)	963 (54.5%)	794 (55.3%)	780 (54.7%)
Darbepoetin alfa, n (%)	482 (27.4%)	520 (29.4%)	377 (26.3%)	403 (28.2%)
Methoxy polyethylene glycol-epoetin beta, n (%)	311 (17.7%)	283 (16.0%)	265 (18.5%)	244 (17.1%)
Baseline ESA dose, U/kg/wk^f^				
N	1,735	1,751	1,427	1,425
Mean ESA dose (SD)	116.6 (109.5)	111.7 (108.6)	113.3 (105.5)	109.4 (110.6)
≤90 U/kg/wk, n (%)	913 (52.6%)	963 (55.0%)	767 (53.7%)	806 (56.6%)
>90 and <300 U/kg/wk, n (%)	720 (41.5%)	691 (39.5%)	584 (40.9%)	541 (38.0%)
≥300 U/kg/wk, n (%)	102 (5.9%)	97 (5.5%)	76 (5.3%)	78 (5.5%)

Abbreviations: AVF, arteriovenous fistula; AVG, arteriovenous graft; BMI, body mass index; CHF, congestive heart failure; ESA, erythropoiesis-stimulating agent; IV, intravenous; N/A, not applicable; NYHA, New York Heart Association; VAT, vascular access thrombosis; wk, week.

a Cardiovascular disease includes coronary artery disease, myocardial infarction, stroke, and heart failure.

b Handling of the partial date of long-term dialysis initiated: If day was missing, day was set to the 15th of the month. If month was missing, month and day were set to July 1. If year was missing, date was missing. Years since long-term dialysis initiated were calculated based on date of long-term dialysis initiated and date of Screening Visit 1.

c Initiation of chronic maintenance peritoneal dialysis or hemodialysis within 16 weeks before screening.

d Populations include patients with both AVF and AVG and a tunneled dialysis catheter.

e No baseline ESA use data are reported for 189 patients in the vadadustat group and 189 patients in the darbepoetin alfa group in the safety population. Not all patients in the safety population were on a currently maintained ESA therapy at the initiation of the INNO_2_VATE clinical program. Current ESA therapy was not included in the inclusion criteria for the incident dialysis-dependent chronic kidney disease trial.

f ESA doses are converted to IV epoetin equivalent U/kg/wk: darbepoetin alfa to IV epoetin is 1:200; methoxy polyethylene glycol-epoetin beta to IV epoetin is 1:220; subcutaneous epoetin to IV epoetin is 1:1.25.

### First and Recurrent VAT Events

During 6,468 PY of follow-up, there were 426 positively adjudicated VAT events in 266 individual patients, including 146 randomized to vadadustat and 120 randomized to darbepoetin alfa (Table 2). Corresponding first event rates were 4.79 and 3.86 per 100 PY, respectively, for a rate difference between the vadadustat and darbepoetin alfa groups of 0.94 (95% CI, −0.10 to 1.97) and HR 1.27 (95% CI, 0.99-1.62) (Table 2). Of the 266 patients who had VAT events, 187 patients had 1 event, 51 had 2 events, and 28 had ≥3 events (Table 3). When considering both first and recurrent events, VAT rates were 6.58 and 6.59 per 100 PY for the vadadustat and darbepoetin alfa groups, respectively (rate difference, −0.01; 95% CI, −1.26 to 1.24; and HR, 1.00; 95% CI, 0.83-1.21) (Table 2).

**Table 2:** Follow-up Time, VAT Event Counts, Proportions, Rates, and Comparative Event Measures

Time to First VAT Event Analysis						
Exposure Group	N	Follow-up Time (PY)	No. of Events	Event Rate No./100 PY	Rate Difference No./100 PY (95% CI)	Hazard Ratio (95% CI)
**Safety analysis set**						
Vadadustat	1,947	3,047	146	4.79	0.94 (−0.10 to 1.97)	1.27 (0.99-1.62)
Darbepoetin alfa	1,955	3,112	120	3.86		

Time to First and Recurrent VAT Event Analysis						
Exposure Group	N	**Follow-up Time (PY)**	**No. of Events**	**Event Rate** **No./100 PY**	**Rate Difference** **No./100 PY (95% CI)**	**Hazard Ratio (95% CI)**
**Safety analysis set**						
Vadadustat	1,947	3,222	212	6.58	−0.01 (−1.26 to 1.24)	1.00 (0.83-1.21)
Darbepoetin alfa	1,955	3,246	214	6.59		

Abbreviations: PY, patient-years, VAT, vascular access thrombosis.

**Table 3:** Number of VAT Events per Patient

	Safety Analysis Set		Baseline Dialysis Type					
			Patients With AVF or AVG		Patients With AVF		Patients With AVG	
	Vadadustat N = 1,947	Darbepoetin Alfa N = 1,955	Vadadustat N = 1,525	Darbepoetin Alfa N = 1,524	Vadadustat N = 1,365	Darbepoetin Alfa N = 1,344	Vadadustat N = 160	Darbepoetin Alfa N = 180
Patients with any VAT event, n (%)	146 (7.5)	120 (6.1)	127 (8.3)	105 (6.9)	79 (5.8)	62 (4.6)	48 (30.0)	43 (23.9)
Total no. VAT events	212	214	188	191	117	94	71	97
1	107	80	93	69	58	49	35	20
2	26	25	21	22	13	8	8	14
3	6	7	6	7	4	2	2	5
4	5	2	5	2	3	1	2	1
5	0	1	0	1	0	0	0	1
6	1	1	1	0	0	0	1	0
7	0	0	0	0	0	0	0	0
8	0	1	0	1	0	1	0	0
9	1	0	1	0	1	0	0	0
10	0	0	0	0	0	0	0	0
11	0	1	0	1	0	1	0	0
12	0	1	0	1	0	0	0	1
13	0	1	0	1	0	0	0	1

Abbreviations: AVF, arteriovenous fistula; AVG, arteriovenous graft; VAT, vascular access thrombosis.

### Sensitivity Analyses

We conducted sensitivity analyses restricting the safety population to patients with an AVF or AVG at baseline (Table S1). Results in this restricted population were consistent with those seen in the full safety population (rate difference, 0.98; 95% CI, −0.24 to 2.21; and HR, 1.25; 95% CI, 0.97-1.63; Table 4). Results stratified by vascular access type are shown in Table 4 and Table S1. These results were broadly consistent with the main analyses, albeit with reduced sample size and precision. Although there were some changes in vascular access type, patients tended to remain on the same type of vascular access following VAT events, irrespective of treatment group (Table S2).

**Table 4:** Follow-up Time, VAT Event Counts, Proportions, Rates, and Comparative Event Measures in Patients With AVF or AVG at Baseline

Time to First VAT Event Analysis						
Exposure Group	N	Follow-up Time (PY)	No. of Events	Event Rate No./100 PY	Rate Difference No./100 PY (95% CI)	Hazard Ratio (95% CI)
**Patients with AVF or AVG at baseline**						
Vadadustat	1,525	2,410	127	5.27	0.98 (−0.24 to 2.21)	1.25 (0.97-1.63)
Darbepoetin alfa	1,524	2,450	105	4.29		
**Patients with AVF at baseline**						
Vadadustat	1,365	2,165	79	3.65	0.83 (−0.23 to 1.90)	1.33 (0.95-1.86)
Darbepoetin alfa	1,344	2,201	62	2.82		
**Patients with AVG at baseline**						
Vadadustat	160	245	48	19.63	2.33 (−5.26 to 9.91)	1.30 (0.86-1.97)
Darbepoetin alfa	180	249	43	17.30		

Time to First and Recurrent VAT Event Analysis						
**Exposure Group**	**N**	**Follow-up Time (PY)**	**No. of Events**	**Event Rate** **No./100 PY**	**Rate Difference** **No./100 PY (95% CI)**	**Hazard Ratio (95% CI)**
**Patients with AVF or AVG at baseline**						
Vadadustat	1,525	2,567	188	7.32	−0.1 (−1.59 to 1.38)	0.99 (0.81-1.21)
Darbepoetin alfa	1,524	2,571	191	7.43		
**Patients with AVF at baseline**						
Vadadustat	1,365	2,268	117	5.16	1.00 (−0.26 to 2.26)	1.27 (0.96-1.67)
Darbepoetin alfa	1,344	2,261	94	4.16		
**Patients with AVG at baseline**						
Vadadustat	160	299	71	23.75	−7.51 (−15.85 to 0.83)	0.78 (0.57-1.07)
Darbepoetin alfa	180	310	97	31.26		

Abbreviations: AVF, arteriovenous fistula; AVG, arteriovenous graft; PY, patient-years; VAT, vascular access thrombosis.

## Discussion

In this secondary analysis of the phase 3 INNO_2_VATE trial program, which included patients with DD-CKD and CKD-related anemia randomized to treatment with either vadadustat or darbepoetin alfa, the rate of first or recurrent VAT was similar between these 2 treatments. The VAT event data presented here were reported by site investigators and positively adjudicated by an independent EAC blinded to patient treatment assignments. The results were robust to several sensitivity analyses, including restriction to patients with documented peripheral vascular access at baseline, or when restricting further to those with an AVF or AVG.

Patients with kidney failure receiving hemodialysis depend on a functional vascular access for their regularly scheduled hemodialysis procedures.^23^ Occurrence of a VAT event almost always disrupts a scheduled hemodialysis session, which can lead to complications related to volume overload (including hospitalized heart failure) and electrolyte disturbances (including hyperkalemia).^24^^,^^25^ For patients of longer dialysis vintage, vascular access sites can be exhausted, obligating them to transition to peritoneal dialysis (which may have been contraindicated or previously not preferred and may be unsuccessful, particularly in patients with little or no residual kidney function) or to depend on a “destination” catheter, unless urgent kidney transplantation is feasible. Finally, even when vascular access patency can be restored, some patients experience complications of pharmacological (thrombolysis) or mechanical (thrombectomy) interventional procedures, including pulmonary embolism and infection. Thus, maintaining patency of hemodialysis vascular access should be an objective of any intervention deployed in hemodialysis.

The INNO_2_VATE trials established the noninferiority of vadadustat to darbepoetin alfa, an ESA with an established cardiovascular risk profile. In the current study, the rates of first or recurrent VAT in vadadustat-treated patients were compared with the rates in patients treated with darbepoetin alfa. Darbepoetin alfa came to market based on comparative trials that focused on hemoglobin correction or maintenance, as well as a phase 4, placebo-controlled outcomes trial conducted in patients with anemia and diabetes mellitus who had advanced, but not DD-CKD, which reported only 7 VAT events as adverse events (4 in the darbepoetin alfa group and 3 in the placebo group).^9^ In 2 phase 3 trials of peginesatide, a peptide-based ESA that was tested against epoetin, the proportion of patients who had a “complication related to vascular access” was similar between treatment groups (18.1% [peginesatide] vs 19.7% [epoetin]).^26^ In a randomized trial of 2,141 patients on hemodialysis in the United Kingdom comparing a higher-dose, proactive to a reactive, lower-dose iron treatment regimen (referent), investigators observed no significant difference in VAT events as (nonadjudicated) treatment-emergent adverse events (HR, 1.15; 95% CI, 0.96-1.38).^27^

Development programs of HIF-PHIs have reported variable details of VAT events from phase 3 trials in patients with DD-CKD. The first drug in this class to be subjected to a large phase 3 cardiovascular outcomes program was roxadustat, for which 4 smaller outcomes trials (PYRENEES, HIMALAYAS, SIERRAS, and ROCKIES) of roxadustat versus an ESA were first reported individually^28^, ^29^, ^30^, ^31^ and then synthesized and meta-analyzed in a subsequent publication.^32^ Among 2,354 patients randomized to roxadustat and 2,360 randomized to an ESA, the VAT rates reported as treatment-emergent adverse events were 5.7 and 3.9 events per 100 PY, respectively. Data on first or recurrent events were not reported.

The ASCEND-D trial randomized 2,964 patients with DD-CKD and CKD-related anemia to open-label daprodustat or an ESA comparator; in the intent-to-treat analysis, 162 (10.9%) and 195 (13.2%) patients had a first VAT event, respectively, which were analyzed together with 17 and 14 deep venous thrombosis and 6 and 6 pulmonary embolism events, respectively, yielding an HR of 0.84 (95% CI, 0.69-1.02).^33^ In a post hoc analysis of VAT events in the ASCEND-D trial, a first adjudicated VAT event occurred in 164 (11.0%) and 201 (13.6%) patients in the daprodustat and ESA groups, respectively (HR, 0.80; 95% CI, 0.65-0.98 and rate ratio of recurrent VAT events, 0.80; 95% CI, 0.63-1.01).^34^

The vadadustat global phase 3 program included 4 randomized controlled trials: 2 trials in patients with DD-CKD (INNO_2_VATE) and 2 trials in patients with non–DD-CKD (PRO_2_TECT). Vadadustat met the prespecified noninferiority criteria for cardiovascular safety in the primary analysis of the INNO_2_VATE trials (HR, 0.96; 95% CI, 0.83-1.11) but not in the PRO_2_TECT trials (HR, 1.17; 95% CI, 1.01-1.36). The trials encompassing the vadadustat phase 3 program in hemodialysis had previously reported on the cumulative incidences of AVF site complication and AVF thrombosis as adverse events, and trials were separated in incident versus prevalent dialysis patient populations.^21^ Hence, we conducted the present study to provide more detailed data and comparisons from the program. Although the first VAT event rate was numerically higher in the vadadustat group than in the darbepoetin alfa group, the HR for a first VAT event was 1.27 (95% CI, 0.99-1.62), demonstrating that statistically, first VAT rates were not different between treatment groups. However, the power to detect marginal differences in VAT rates may have been limited in this study as can be gleaned from the width of the CI. When both first and recurrent VAT events were considered, however, VAT rates were nearly identical between treatment groups with reasonably tight confidence limits.

Strengths of this study include the prospective collection of vascular access complications and adjudication of VAT events by an EAC blinded to treatment assignment. Additionally, this study included rigorous statistical testing wherein we considered time to first VAT event as well as time to first or recurrent VAT event. We conducted these analyses for the overall safety population as well as subsets of patients on hemodialysis and those who had peripheral vascular access (AVF or AVG) at trial enrollment. Limitations of the study include the fact that the parent INNO_2_VATE trials were designed principally to determine cardiovascular safety. At study onset, VAT rates were relatively low; the power to detect modest but clinically meaningful differences in VAT rates (vadadustat vs darbepoetin alfa) was probably low.

There are multiple reasons for hemodialysis vascular access failure. Differences in surgical technique, diameter and integrity of arterial inflow and venous outflow, intimal hyperplasia at the venous anastomosis (in the case of AVGs), ambient blood pressure, the frequency and severity of (intradialytic) hypotensive episodes, acute and chronic inflammation, and other factors unrelated to thrombosis can contribute to what we refer to as “vascular access thrombosis.” Thus, it is theoretically possible that treatment with 1 of 2 drugs with vastly differential effects on platelets or the coagulation cascade might not result in differential rates of vascular access failure.

In conclusion, in patients with DD-CKD and CKD-related anemia receiving hemodialysis, first VAT rates were numerically higher in patients treated with vadadustat than in patients treated with darbepoetin alfa but statistically not different. When considering both first and recurrent VAT events, the rates were similar between treatment groups.
