# Supplementary material for: Vascular Access Thrombosis Events in Patients With Dialysis-Dependent CKD Treated With Vadadustat or Darbepoetin Alfa: The INNO2VATE Trial Program
Source: Kidney Med. 2025 Mar 19;7(5):100997. doi: 10.1016/j.xkme.2025.100997 (PMC12051538; doi:10.1016/j.xkme.2025.100997)
Supplement: Supplementary File (PDF) — Table S1-S2 [file mmc1.pdf]

Table S1. Selected Demographic, Clinical, and Laboratory Characteristics of Patients at Baseline (Safety Population With Baseline Dialysis Type as Either AVF or AVG)

Characteristic	AVF at Baseline		AVG at Baseline	
	**Vadadustat** N = 1365	**Darbepoetin alfa** N = 1344	**Vadadustat** N = 160	**Darbepoetin alfa** N = 180
**Demographics and Baseline Characteristics**				
Age, year, mean (SD)	57.8 (14.0)	58.3 (13.7)	60.2 (12.2)	60.6 (13.5)
Male, no. (%)	787 (57.7)	794 (59.1)	79 (49.4)	78 (43.3)
Race, no. (%)				
Asian	43 (3.2)	53 (3.9)	3 (1.9)	7 (3.9)
Black or African American	298 (21.8)	304 (22.6)	78 (48.8)	80 (44.4)
White	941 (68.9)	884 (65.8)	74 (46.3)	89 (49.4)
Not reported	37 (2.7)	38 (2.8)	1 (0.6)	2 (1.1)
Other	46 (3.4)	65 (4.8)	4 (0.3)	2 (0.1)
Region, no. (%)				
United States	797 (58.4)	784 (58.3)	145 (90.6)	147 (81.7)
Europe	216 (15.8)	232 (17.3)	3 (1.9)	10 (5.6)
Rest of World	352 (25.8)	328 (24.4)	12 (7.5)	23 (12.8)
Hemoglobin (g/dL), mean (SD)	10.2 (0.9)	10.2 (0.9)	10.0 (0.8)	10.1 (0.8)
BMI (kg/m^2), mean (SD)	28.4 (7.1)	28.3 (6.9)	29.0 (6.9)	29.8 (7.6)
NYHA Functional Class, n (%)				
Class 0 (no CHF) or I	1190 (87.2)	1170 (87.1)	143 (89.4)	151 (83.9)
II or III	175 (12.8)	174 (12.9)	17 (10.6)	29 (16.1)
Disease history, no. (%)				
Diabetes mellitus	721 (52.8)	733 (54.5)	101 (63.1)	102 (56.7)
Cardiovascular disease*	663 (48.6)	704 (52.4)	97 (60.6)	111 (61.7)
**Dialysis-related characteristics**				
Time since dialysis initiation†				

Mean (SD)	4.036 (4.0658)	3.966 (4.0227)	4.789 (4.9548)	4.513 (4.5084)
Median	2.795	2.637	3.273	3.370
Q1, Q3	1.175, 5.681	1.172, 5.536	1.565, 5.989	1.484, 6.205
Incident dialysis patient[‡], no. (%)	99 (7.3)	117 (8.7)	7 (4.4)	6 (3.3)
Dialysis modality, no. (%)				
Hemodialysis	1355 (99.3)	1340 (99.7)	160 (100.0)	180 (100.0)
Peritoneal dialysis	10 (0.7)	4 (0.3)	0	0
Vascular access, no. (%)				
Temporary catheter	0	0	0	0
Tunneled dialysis catheter	2 (0.1)[§]	1 (0.01)[§]	0	1 (0.06)[§]
Other	0	0	0	0
Baseline ESA use[ǁ]				
no.	1279	1252	157	175
Epoetin, no. (%)	730 (57.1)	700 (55.9)	64 (40.8)	80 (45.7)
Darbepoetin alfa, no. (%)	329 (25.7)	344 (27.5)	48 (30.6)	59 (33.7)
Methoxy polyethylene glycol-epoetin beta, no. (%)	220 (17.2)	208 (16.6)	45 (28.7)	36 (20.6)
Baseline ESA dose, U/kg/week[¶]				
no.	1271	1250	156	175
Mean ESA dose (SD)	111.63 (105.0)	108.16 (109.2)	126.86 (108.2)	117.95 (120.2)
≤90 U/kg/week, no. (%)	697 (54.8)	713 (57.0)	70 (44.9)	93 (53.1)
>90 and <300 U/kg/week, no. (%)	507 (39.9)	472 (37.8)	77 (49.4)	69 (39.4)
≥300 U/kg/week, no. (%)	67 (5.3)	65 (5.2)	9 (5.8)	13 (7.4)

*Cardiovascular disease includes coronary artery disease, myocardial infarction, stroke, and heart failure.

[†]The handling of the partial date of chronic dialysis initiated: If day is missing, day is set to 15th of the month. If month is missing, month and day are set to July 1. If year is missing, date is missing. Years since chronic dialysis initiated are calculated based on date of chronic dialysis initiated and date of Screening Visit 1.

[‡]Initiation of chronic maintenance peritoneal dialysis or hemodialysis within 16 weeks prior to screening.

[§]Populations include patients with both AVF and AVG and a tunneled dialysis catheter.

[‖]No baseline ESA use data are reported for 189 patients in the vadadustat group and 189 patients in the darbepoetin alfa group. Not all patients in the safety population were on a currently maintained ESA therapy at the initiation of the $INNO_2VATE$ clinical program. Current ESA therapy was not included in the inclusion criteria for the NCT02865850 clinical trial.
[¶]ESA doses are converted to IV epoetin equivalent U/kg/week: darbepoetin alfa to IV epoetin is 1:200; methoxy polyethylene glycol-epoetin beta to IV epoetin is 1:220; subcutaneous epoetin to IV epoetin is 1:1.25.
AVF, arteriovenous fistula; AVG, arteriovenous graft; BMI, body mass index; CHF, congestive heart failure; ESA, erythropoiesis-stimulating agent; IV, intravenous; NYHA, New York Heart Association.

Table S2. Type of Vascular Access After VAT Events (Safety Population)

**Category**	**Vadadustat** N = 1947	**Darbepoetin Alfa** N = 1955	**Total** N = 3902
Total VAT events, no.	212	214	426
Total events in patients with AVF, no. (%)*	120 (56.6)	87 (40.7)	207 (48.6)
Type of vascular access after VAT event in patients with AVF, no. (%)†			
AVF	86 (71.7)	64 (73.6)	150 (72.5)
AVG	0	0	0
Temporary (i.e., nontunneled dialysis) catheter	10 (8.3)	10 (11.5)	20 (9.7)
Tunneled dialysis catheter	13 (10.8)	6 (6.9)	19 (9.2)
Other	3 (2.5)	0	3 (1.5)
Missing	8 (6.7)	7 (8.1)	15 (7.3)
Total events in patients with AVG, no. (%)*	77 (36.3)	100 (46.7)	177 (41.6)
Type of vascular access after VAT event in patients with AVG, no. (%)‡			
AVF	0	0	0
AVG	60 (77.9)	84 (84.0)	144 (81.4)
Temporary (i.e., nontunneled dialysis) catheter	1 (1.3)	2 (2.0)	3 (1.7)
Tunneled dialysis catheter	7 (9.1)	5 (5.0)	12 (6.8)
Other	0	2 (2.0)	2 (1.1)
Missing	9 (11.7)	7 (7.0)	16 (9.0)

*Patients with specified access type prior to and closest to time of event. Percentage calculated based on total VAT events.
†Percentage calculated based on events in patients with AVF.
‡Percentage calculated based on events in patients with AVG.
AVF, arteriovenous fistula; AVG, arteriovenous graft; VAT, vascular access thrombosis.
